# A Monograph on Ovarian Tumors

**Published:** 1857-05

**Authors:** T. M. Tweed

**Affiliations:** North Liberty, Ohio


### SELECTIONS.

*A Monograph on Ovarian Tumors; with an extended view of Ovariotomy as a means of cure.* By T. M. Tweed, M. D., of North Liberty, Ohio. (*Continued from page* 489.)

The Treatment of Ovarian Dropsy.—In no disease has the application of medicine hitherto been of so little avail as in the one under consideration. It has been acknowledged by many, and, indeed, by nearly all, who have attempted its cure, "that medicine has no power over it." Dr. Hunter says, that "the ovarian dropsy is an incurable disease; and that the patient will have the best chance of living longest under it, who does the least to get rid of it."

Dr. Elliotson says, "that if any medicine does good in these cases, it is iodine." Again, "if iodine did not exist, I would not use any medicine at all in these cases, for excepting it I never found any, of whatever kind, to do the least good."

Dr. Blundell, speaking of purgatives, diuretics, mercurials, etc., in this disease, says, "they do no good. I will not venture to say you are not justified in making gentle attempts with these remedies; but experience shows that from these medicines so little good is to be obtained that in attempts like these the constitution ought not to be injured." And lastly, Burns states that "medicine has as much power over these cystic tumors as it has over the configuration of the patient's nose."

If these opinions alone are regarded, the attempt to cure ovarian dropsy would appear absurd and ridiculous. But although this want of power in medicine is seen in many cases, in some it does produce benefit; and although it may not establish a cure, it may so retard the progress of the disease as to enable the patient to live in comparative comfort for some years.

Our observations upon the treatment of this disease will be comprised under two distinct heads:

1st. The Palliative Treatment; 2d, the Radical Cure.

The Palliative Treatment of ovarian dropsy consists in the employment of Medical and Surgical remedies. It is said to have been *cured* under various plans of treatment, the success varying according to the age, health, character of the patient, and the longer or shorter duration of the disease.

If enlargement of the ovary arises from inflammatory action, the enlarged organ can be felt distinctly between the vagina and rectum, and is very painful on pressure. In this case a strict antiphlogistic treatment is to be pursued. Local blood-letting is very important; this is to be effected not as usually prescribed, viz: by cupping the loins, or by the application of leeches to the vulva, but what is more effectual, by the application of leeches to the tumor itself through the rectum. The bowel is to be washed out by a copious enema; then by placing the leeches in a long glass tube, the upper end of which is perforated by a number of holes, the mucous membrane bulges through these perforations on introducing it into the bowel, and the leeches usually fix themselves to it. Care is necessary in introducing this instrument, and when it arrives at the diseased ovary it produces pain, and ought not to be pushed further. Large quantities of blood may be taken in this way, and the application ought to be repeated every fourth day until the inflammatory symptoms subside.

This local depletion is to be further assisted by calomel and opium and saline purgatives. Blisters and leeches ought to be applied to the tumor when above the pubis, when pain or uneasiness is present. Dr. Ashwell regards this antiphlogistic plan as very efficacious in the early stages of the disease. He says, "I have sometimes found local bleedings by leeches followed by repeated blisters (kept on only for a few hours,) and succeeded by linseed poultices for several days, have not only retarded further growth, but have diminished the absolute bulk of some incipient ovarian tumors."

When the tumor in the posterior wall of the vagina gives any sense of fluctuation, it ought to be punctured. This should be done with a curved trocar, where the fluctuation is most distinct.

The tumor in this position often draws down the fundus of the uterus, so as to produce retroversion of that organ; in such cases there is difficulty in evacuating the urine, and sometimes there is retention; if so the catheter must be used. Constipation is almost always present, producing pain, to overcome which, aperients are necessary.

In spite of all our endeavors the tumors may increase and occupy the cavity of the abdomen; and it is in this position we are more frequently called upon to treat it.

In this stage, also, the antiphlogistic plan of treatment has been advised, and was the one followed by Mr. Abernethy, "in order to reduce any inflammatory symptoms, and produce, if possible, absorption of the contents of the sac." Leeches applied to the tumor, followed by small and repeated blisters, have been recommended. This plan is very efficacious in removing any pain that may be present; and the constant irritation may be beneficial in removing the contents of the tumor; indeed, Dr. Bernott, of Cork, relates an instance where an ovarian dropsy was entirely cured by the constant application of counter-irritation in the form of a large seton applied over the tumor. Small blisters, also, have been used, and said to have been very beneficial to these swellings.

The most powerful remedies in this disease and those which seem to have the most influence over it, appear to be iodine and the liquor potasse, We have already stated that in the opinion of Dr. Elliotson, iodine is the only remedy we would use; as all others under his observation had entirely failed. He says, "I have seen cases diminish and some apparently cured by this remedy." Dr. Seymour also speaks very highly of it and gives cases illustrative of its remedial powers; he thinks it acts by producing suppuration of the cyst with adhesion of it to some of the neighboring organs and the discharge of its contents. The constitutional symptoms before these desirable events take place, are frequently very severe and often destroy the patient. Dr. Seymour remarks in one case under the action of this remedy "the tumor appeared to gradually grow softer; at length very violent constitutional symptoms arose, tremblings, great distress of mind, and lowness of spirit; to which succeeded the symptoms of internal suppuration, a very quick pulse, tongue brown and dry, rigors, followed by profuse sweats. At the expiration of a fortnight, the patient began to pass purulent matter by the rectum and vagina of various consistence and intolerable odor; this passed daily for some weeks and the patient recovered.'' In most of the recorded cases where some of the preparations of iodine have been used with success, as in Dr. Elliotson's, A. T. Thompson's and others, the tumor itself has been found to become softer on its surface, adhesions have taken place to some of the neighboring viscera, ulcerations have occurred between their walls, and the contents of the cysts have been injected into their cavities to be discharged at their natural outlets.

The desired objects in the use of this remedy appear to be suppuration of the cyst, and the discharge of its contents; but we are not always able to secure them; the inflammation may rise too high and induce a fatal result, or no effects at all will be perceived by its application; but in the majority of cases iodine acts more by inducing suppuration of the cyst than by any absorbent powers it may possess.

Mr. Jeafferson (*Med. Gaz.*, *Sept.*, 1844,) says, "I have also had several opportunities of witnessing the *gradual softening* of ovarian tumors under the use of iodine, when I have not been able to learn the ultimate termination of the case. This softening process on the tumor appears to be the effect of these remedies; they do not, however, possess much, if any influence in promoting its direct absorption. What is the precise *modus operandi* it is not easy to decide.''

From these remarks we may perceive that iodine, as a remedy in this disease, requires great care in its administration; that if any unpleasant effects are produced it should be discontinued for a time, and a return to its use should be careful and guarded.

The iodide of potassium is the remedy chiefly employed in ovarian dropsy, and possesses the advantage of combining the iodine and potash. When the system is fully under its influence, there are disagreeable sensations about the nose, coryza is present and an eruptive acne is observed about the shoulders. The syrup of the iodide of iron is an effectual and pleasant remedy for delicate females. When dyspepsia is present the iodide of potassium is given in doses of five, increased to twenty grains in some bitter infusion, two or three times a day; or it may be given with a purgative when constipation is troublesome.

Some patients are unable to take iodine in any of its forms on account of its action being very quickly displayed in their system; a good substitute in such cases is the liquor potasse. This medicine given in as large doses as the stomach can bear (small beer or table ale is the best vehicle on account of its efficiently disguising the taste,) has been very successful under the direction of Sir. B. Brodie in removing scrofulous and steatomatous tumors; it is found also to act in a similar way to iodine in ovarian dropsy. Dr. Seymour states that this remedy has been used in ovarian disease; that the general health has appeared to be often greatly improved under its use, and the formidable disease itself is reported to have disappeared under its employment. "The liquor potasse in such cases, appears to act by inducing suppuration in the cysts which is afterward discharged, adhesions having been formed with the neighboring viscera. In this respect its action resembles that of iodine, and is contraindicated when increased vascular action is present; and in fact it is in the leucophlegmatic habit of body that it appears to be most applicable, whether as a curative or only as a palliative remedy."

Dr. Warren relates a case in his work on tumors, where this remedy produced softening of the tumor and a discharge of purulent matter by rectum, with a perfect cure of the patient.

The late Dr. Hamilton of Edinburgh, proposed a plan of treatment in ovarian dropsy, which, under his management, is said to have been very successful. It consisted of moderate bandaging, percussion on the tumor, and small doses of the muriate of lime. The percussion could either be made by means of the fingers, or by an instrument consisting of five balls attached by rods at right angles to the handle so as to somewhat resemble the hand and five fingers. I will give his own description of his mode of treatment; he says, "adverting to the effects of percussion and pressure in chronic rheumatism, and knowing the influence of the continued use of the muriate of lime in indolent glandular swellings, I was led to the trial of these several means as being at any rate perfectly safe. I advised, therefore, that moderate and equable pressure of the abdomen should be made by means of a suitable bandage; that the large part should be subjected twice a day to gentle percussion; and that a course of small doses of muriate of lime should be continued for at least for several months. When pain or tenderness was experienced on the ovary being pressed upon, I recommend the daily use of the warm bath. This plan of treatment has been much more successful than I had anticipated; in seven cases in which it has been used, the enlargement has so completely subsided that it is no longer tangible. There could be no mistake in the majority of these cases, not only because the size of the diseased ovary was very considerable, the fluctuation was distinct and all the ordinary characteristics were well marked, but also because the nature of the affection had been previously ascertained by some of the most experienced practitioners in London. Previously to the diminution of bulk in all the successful cases, it is proper to add that the circumscribed enlargement of the ovary has invariably become soft. This change was so remarkably obvious in the first of the successful cases, that the indentation of the patient's fingers upon it was similar to what occurs in anasarca, although it had been previously incompressible, as the tumor extended as high as the right hypochondrium. This important change was first perceived by the patient herself."

This plan has not been so successful in the hands of English practitioners as in those of Dr. Hamilton. The question arises, whether the plan has been really tried, or only partially put in practice. It is to be feared that the latter has been the case; for when we consider the difficulties which arise in treating a chronic case, where the improvement is very slow, and scarcely perceptible, we cannot wonder that the patients exertions should become relaxed, and the chance of cure abandoned.

Mr. Isaac B. Brown has lately published a plan of treatment, having the same indications as Dr. Hamilton's, although the means by which he intends to secure success are different. His plan consists in evacuating the cyst by tapping, after it has ceased to increase under the use of mercurial remedies and diuretics; and then by applying large pads over the cyst, and bandaging the abdomen very tightly, he endeavors to obtain obliteration of the sac, and consequent cure. He has published several successful cases arising from this treatment. (*Vide, London Lancet*, vol. 1, New Series, page 179.)

He says: "I divide my treatment into constitutional and local, and treatment after tapping.

"1. The constitutional one consists in the administration of mercurials internally as alteratives, and externally by friction over the abdomen, and continued until the gums are slightly, but decidedly affected; and this must be continued for some three weeks. I lay particular stress upon this point; at the same time, diuretics must be given, and after the first week, tonics must be combined with them. The food should consist of light animal diet, and the patient should take daily exercise in the air.

"2. Local treatment. This consists of the careful application of a tight flannel bandage, so as to produce considerable pressure over the tumor. When it is found that the abdominal action has been checked by a positive decrease in the tumor, and a continuation of such decrease, or by a positive non-increase for some weeks, then the cyst should be tapped, and all its fluid evacuated.

"3. Treatment after tapping consists of accurate padding and tight bandaging over the cyst and body, generally for two or three weeks; and the medicines and position ought to be continued for at least six weeks. I would particularly wish to enforce the importance of the after-treatment, as on that depends very much the success or failure of the case."

This plan of treatment has been given to the profession, and apparently sanctioned by a number of successful cases; but we are bound to add, that some of those cases, called and published as successful, have come into other hands; and a highly respectable physician states, that two of Mr. Brown's cases have come under his observation—one died of ovarian dropsy, and on a *post mortem* examination, the cyst was found still to exist as large as before; "the other," he adds, "is still ill; the cyst has refilled, and we were obliged to have recourse to tapping."

This fact reduces considerably the value of Dr. Brown's cases. Again, on referring to the cases themselves, can we, on their recital, confidently assert that they are all cases of ovarian cysts? The real diagnostic marks are not *too clearly* stated, and the fluid evacuated by some, resembles that secreted by the peritoneum.

In a discussion at the Physical Society of Guy's Hospital, where the point was urged, Mr. Brown failed to convince the members that a cystic tumor was present in several of the cases he related to them. And, lastly, we would ask, can the system of salivation, which is an essential part of the treatment, be borne by many, or ought it to be administered in others who are young and healthy? The experience of the heads of our profession are against its administration; and some think "that we are not justified in persevering in a remedy which sometimes produces direful effects upon the constitution, and has so little effect upon the cyst itself."—BLUNDELL.

Pressure properly applied, is undoubtedly the best part of this plan; but this is not original, as Mr. Hamilton and others had advocated its efficacy long before Mr. Brown's treatment was thought of. Besides, pressure can only be applied in a limited degree; for if it be too forcibly made, the circulation becomes interfered with, and it is difficult to be borne. Even if great pressure can be maintained steadily, we are fully aware that it usually fails to obliterate cysts on the *external* parts of the body.

In a cystic tumor of the scalp, the most favorable place for pressure, it rarely obliterates the sac. Of course, we can hardly suppose that the effects of pressure can be of much service, where there is no point of resistance, and where serious consequences may be produced in other organs. Pressure, again, can only be tried with a hope of success, in those cases where the cyst is simple, for it must inevitably fail in the multilocular variety. In the latter case, all that we could hope to accomplish, would be to retard its rapid development.

The application of pressure to the abdomen, produces a resistance to the rapid development of these bodies, and acts in the same way that extensive adhesions would do, in arresting its enlargement, by placing resistance to its increase.

Dr. F. H. Ramsbotham relates a case of this kind, which occurred in the practice of his father.

With all our vigilance and perseverance, the tumor may gradually increase to such a size as to become troublesome by its bulk, and endanger life by its interference with the vital functions. We shall, in this stage of the disease, have to treat the various symptoms arising from pressure. The tumor may encroach upon the stomach, and cause constant vomiting, which may baffle the most skillful and varied treatment. That this is the result of mechanical pressure, is proved by many cases. It is true the functions of the stomach may become permanently diseased, and even organic changes occur, but this is not usually the case. In one instance seen by Dr. Lee, the vomiting ceased immediately after tapping; so that without this reduction in size, the ordinary treatment by effervescing salines, hydrocyanic acid, cresote, sinapisms, and blisters, would be of no avail. Dispnea is also a very frequent complication of this stage of the disease. It may be partially relieved by position, but its permanent remedy is the reduction of the sac.

The kidneys are, also, very frequently interfered with : the pressure of the enlarged ovarian prevents the proper secretion from taking place. Here some strongly recommended diuretics, but they can do no good, the cause being mechanical not functional. Remove the pressure, and the kidney gives out its secretion, natural in quantity and consistence. This fact was well illustrated in a case already noticed, where the secretion of urine was greatly diminished during the distension of an enormous cyst, but was instantly restored to its natural quantity and quality, after the pressure had been removed by tapping. Suppression again took place, on the enlargement of the tumor, and resisted every diuretic.

Diuretics are valuable, when ascites exist as a complication, but should never be used where the pressure is the cause of suppression; they are also useful where there is œdema of the ankles and eyelids.

Having now presented the principal remedies and plans of treatment, recommended by the best practitioners, and perceiving how little the disease is amenable to medicine, we pass to the consideration of paracentesis as a means of palliation and cure, adopted by many respectable authorities.

Paracentesis.—Practitioners generally have a great dislike to a recourse to this mode of palliating the disease. Experience teaches them that, in a majority of cases, the relief obtained is but temporary, the cyst rapidly refilling, leaving the patient in a worse condition than before. There are cases on record, however, in which this remedy has been followed with perfect success. Dr. A. T. Thompson reports a case in which tapping was performed fourteen times, and the patient recovered.

The operation, in itself, is considered one of the most simple in surgery. The patent generally sits in a chair, or on the side of a bed, with a broad piece of flannel covering the abdomen, the ends being slipped up, in order to adapt the pressure equally to the upper and lower portions of the abdomen. These ends are placed one within the other, and drawn tightly by assistants; a small opening is made in the flannel anteriorly, through which the trocar passes. This instrument may be introduced at once, or the skin may be first divided (which is the most usual way,) by a bistoury or lancet; or, lastly, the latter may be carried directly into the sac, and a blunt-pointed trocar and canula may be introduced into the opening. Gradual pressure is to be made with the bandage, in order that the contents of the cyst may be evacuated, and, also, to secure the patient from fainting, and protect the viscera, which would otherwise be left unsupported, by the withdrawal of the fluid. Immediately after the operation, the patient often feels faint, and sometimes syncope takes place, especially if the patient be not kept up upon the abdomen. This has sometimes, but rarely, been fatal; usually the patient recovers quickly, and feels great relief; instead of dyspnea, a feeling of distension and fear of suffocation, there is distinct calm; the lungs perform their office, and the distress ceases.

After tapping, the disappearance of the tumor is sometimes entire, at others only partial. This depends upon the character of the cyst. If it be simple it almost entirely disappears; but if multilocular the patient is surprised to find large hardened masses still remaining in the abdomen. In a few days she feels herself to become more distended, and from this time the abdomen gradually enlarges until it attains the same or a larger size than before the operation.

There are several points worthy of notice in the operation of paracentesis.

1st. The operator should correctly ascertain the most prominent part of the tumor, and the situation of the space where fluctuation is most distinct. That portion mid way between the umbilicus and pubis in the linea alba is the most appropriate; but in the multilocular variety, on examination and percussion, distinct hard masses may be found there, which ought particularly to be avoided; for if the trocar is introduced into them, the fluid contents of the sac will not be drawn off, and the patient will be subjected to the danger of an inflammatory attack.

2d. The patient ought always to be informed that the actual decrease of the tumor may be very slight, especially if we suspect a cyst of the multilocular variety. In this case, a small cyst giving distinct evidences of fluctuation, may be opened, and only a few ounces of fluid be evacuated, giving only partial relief, and the operator may be compelled to make another puncture.

3d. The trocar should be introduced with a certain degree of justifiable force. A timid surgeon often fails in getting into the cyst, from fear of using too much force. The walls of these tumors are frequently very firm and dense, and if a certain degree of power is not used the sac will be pushed before the trocar and its cavity will not be opened.

4th. All large veins should be avoided.

5th. It is necessary to be particular in the diagnosis, and always to ascertain whether the bladder has been fully evacuated. If any doubt exists the catheter should be introduced before the operation; indeed, this ought to be done in every instance, as we cannot always depend upon the patient's opinions or expressions; for cases have occurred in which the distended bladder and the pregnant womb have each been punctured for cystic dropsy.

Dangers of Paracentesis.—This operation may be performed very many times on the same individual without any bad effects, although it may give only occasional or partial relief to the patient. Several cases are recorded of enormous amounts of fluid being taken away by a great number of operations, through a series of years, some during thirty years, with no marked effects upon the constitution. All readers of surgery are acquainted with the case of "Dame Mary Page," who in sixty-seven months, was tapped sixty-six times, and discharged 240 gallons of water without ever repining at her case or ever fearing the operation.

But there have been larger quantities of fluid withdrawn, and the patient has survived even a greater number of tappings than good Dame Mary Page. In the celebrated case related by M. Martieau of Norwich, there were 6831 pints or 13 hogsheads of fluid withdrawn from an ovarian cyst during eighty different operations. (*Vide. Philos. Trans., Vol.* 74, *page* 471.)

Dr. Buckner tapped a patient upwards of twenty times during a period of two years. She at last died of inflammation of the cyst which was found to be multilocular and of large size.

In most of the medical journals of the day, may be found some of these extraordinary cases; and it is no slight encouragement for the afflicted, to be made acquainted with the facts. But we must not blind our eyes with the *exceptions* and forget the *rule*, for these cases are singular and therefore recorded, while the majority die in much less time and are buried in forgetfulness.

Dr. Blundell's practical observations on this point are well worthy the profoundest attention. He remarks "although women do live now and then to undergo these frequent tappings, yet they more generally sink; and hence, in ordinary practice, the longer the first tapping can be delayed the better, for there is nothing more unwise than to ground your general practice upon the *exception* to the RULE, though the error is not unfrequently committed."

It is, then, possible for the operation of tapping to be performed, and no danger arise; the patient may not recover from its effects until it is again required; but this is not always, nor indeed generally, the case. Rapid and fatal syncope may follow the operation, or the patient may die from exhaustion, after having rallied for a few days.

The natural tendency of an ovarian tumor, when uninterfered with, is to grow *slowly*; but when the fluid is withdrawn, the pressure which before existed, is taken from the secreting vessels; consequently, re-accumulation of fluid quickly follows, which frequently results in inflammation of the cyst, with rapidly fatal symptoms.

Danger arises from this operation, by the accidental puncture of one of the large vessels, which frequently ramify on the surface of the cyst. Dr. Buckner observes, "I have now seen several *post-mortem* examinations, where these tumors existed, and have observed large vessels, nearly the size of the little finger, ramifying on the sac, and one was placed in such a position that it would have been wounded, had an operation been performed." These large vessels, also, may arise in the omentum, which may be intimately attached to the anterior part of the cyst. This peculiarity occurred in a case in which the operation of ovar'otomy was performed, witnessed by Dr. Lee, who says, "the vessels were as large as those of the dura mater,

The greatest danger to be apprehended after tapping, is the inflammation of the cyst itself, or the peritoneum. This is almost the inevitable termination, at some period or other, of the lives of patients who are subject to the operation. According to my own experience, the cyst itself is the part most usually inflamed. In some cases, a portion of the fluid escapes and acts as a foreign body on the peritoneum; or the trocar may have punctured a mass of cysts, and thus produced inflammation.

The effects of the inflammation, however produced, are alarming; all the symptoms of active fever are found, there is great pain in the abdomen, which becomes tense, and very tympanitic; vomiting ensues, and rapid exhaustion takes place, followed by death.

We have taken pains to collect a number of cases, in which the duration of the disease, after the first tapping, was accurately recorded, and we find that more than one-half of those who died did so within four months, and a moiety of these were only tapped once. Almost all the deaths, after the first operation were attributed to inflammation of the sac, or the peritoneum.

The operation in some instances, cures the cystic tumor. This happens when it is unilocular and simple; but these cases are rare, and, from the facts about to be adduced, we may well dread to perform the operation.

In very many cases, paracentesis can do no good, the tumor being made up of several small cysts; in many others it only affords partial relief; and in some, it actually kills. Dr. Blundell has well said, "Make the best of it, and tapping, after all, is an unsatisfactory sort of remedy; dangerous in scirrho-dropsy; of partial relief in dropsy of many cysts; of no effect where the cystic material is viscid; obnoxious to inflammations, adhesions, suppurations. exhaustions, repetitions, and death, even in cases the most favorable; and the more I have seen of this operation, the more I have felt inclined to whisper to myself, when the surgeon has taken up the instrument, I wish I could do something better."

When is Paracentesis to be Performed?—If the operation is decided upon, it is a matter of no small moment to determine *when* it shall be performed. Three periods have been suggested, each of which has its advocates, and its supposed peculiar advantages.

(1.) When the tumor arises just above the pubis.

(2.) When it occupies the abdomen, but without great distension; and,

(3.) When it presses upon important viscera, and impedes the vital functions.

The operation in the first position is recommended by Dr. Blundell, upon the principle that the surface from which fluid is secreted is small at that period, and that there is a greater chance of a curative process being established.

The operation can be performed easily enough when the tumor is situated between the vagina and rectum, and when the fluctuation is distinct.

Dr. Odge, of Rochdale (*London Medical Gazette, Vol. XXIV,*) gives a case of successful cure by this method. In a similar case, it succeeded for a time ; but the patient took out the canula which was left, and the secretion again returned. In another case operated on in this way, it terminated unsuccessfully, from the cyst being multilocular, and the base of the tumor forming almost a solid substance, although there was distinct fluctuation. Many successful cases might be quoted, and especially such as have been discovered and operated upon, during parturition.

But Dr. Blundell thinks that the tumor might be opened when it is as large as a child's head, and situated above the brim of the pelvis, "Now, supposing our knowledge be sufficient, and our caution great, would it perhaps be impracticable to effect all this, even when the tumor lay above the brim of the pelvis, in the hollow of the illium. For this purpose, might not an opening be made in the abdominal covering, large enough to admit the forefinger like a canula ; and might not the point of the finger be placed on the surface of the ovary, so as to ascertain that no intestine was interposed, and, then, when sure the intestines and bladder are not interposed, might we not pass a very small trocar through the opening, and into the ovary, so as to evacuate the contents, at the very commencement of the disease ?"—*Blundell.*

There can be no doubt that the early evacuation of the fluid, is a desirable proceeding ; and when the tumor can be felt in the vagina, having distinct fluctuation, it ought decidedly to be punctured. The success of this operation has been great, when the disease has complicated labor. From our knowledge of abdominal surgery at the present time, we are aware that a small incision into the peritaneal cavity is not so dangerous as at one time we were led to suppose. But we should be quite certain of the existence of the cystic nature of the tumor, before such an attempt is proceeded with.

Dr. Bright does not approve of this early paracentesis. "It has," says he, " been recommended to have recourse to paracentesis, when the tumor is as large as the uterus at the termination of pregnancy, before the vital functions are impeded, or the distension of the cyst becomes larger. " I conceive," says he, " that the time of the operation has arrived, when the tumor pretty fully occupies a large portion of the abdomen, giving the appearance of pregnancy advanced to the last months, and before any material mischief seems to threaten either the surrounding viscera, or the parietes of the tumor itself ; for there can be little doubt that the forcible distension of the sac, continued beyond a certain limit, will endanger its inner surface, and, perhaps, prove one cause of the ulcerative changes which often take place, and are the source of great constitutional irritation, and death."

And, lastly, many practitioners agree, that the rapid re-filling of the cyst, after the first tapping, is so dangerous, and produces such fatal effects, that they willingly defer the operation until they are compelled to relieve their patient from the severe systoms they suffer—the operation being performed from necessity, not from choice.

We do not think any distinct rule can be laid down, which, would embrace the period of tapping, in all cases of ovarian dropsy. It appears that each individual case has its peculiarities ; that the period of the disease, at which we are called to prescribe, is so various, and the nature of the cysts is so different, that each case ought to be treated individually, according to the tact of the surgeon. When the patient is young and healthy, the plan of puncturing the cyst early, and applying pressure and friction, is, perhaps, the best mode of treatment ; and the employment of iodine internally, so as not to injure the general health, is beneficial. But, when a large, multilocular cyst come under treatment, that will be the best where least is done to the local disease, and the general health supported. In such a case, tapping is injurious, and ought, if possible, to be avoided.

Several other surgical operations upon the cyst itself, have been suggested, with a view to destroy the secretory power of its lining membrane. It has been proposed to make extensive incisions into the cyst, or take portions away ; and cases are recorded by Le Drau and others, of cure by this treatment. Setons have been passed through the walls of these cysts, and tents have been left in openings made into them, for the purpose of producing suppuration and adhesion of their internal parietes. Mr. Key tried these remedies in several instances, but found them to fail in all. Cysts have, also, been injected with irritating fluids, for the same purpose. An aqueous solution of iodine and iodide of potassa, has been highly recommended; and if injecting the cyst is at all justifiable, it is, perhaps, the very best that can be used. Some cases have partially justified the treatment, while in others it has completely failed.

All these plans are now rejected from modern practice, and we think very justly, because the constitutional irritation, following their application, is so great as to be, in most nistances, fatal. At best, when these remedies have done their utmost, the disease is not cured ; fitulous openings remain, and at last the patient dies exhausted.—*Cincinnati Med. Observer.*

